# Research on the Interaction between Tubeimoside 1 and HepG2 Cells Using the Microscopic Imaging and Fluorescent Spectra Method

**DOI:** 10.1155/2014/470452

**Published:** 2014-05-14

**Authors:** Xiaogang Lin, Wenchao Li, Changbin Ye, Xiaozhu Liu, Hao Zhu, Wenbing Peng, Jie Rong

**Affiliations:** Key Laboratory of Optoelectronic Technology and Systems of Ministry of Education of China, Chongqing University, Chongqing 400044, China

## Abstract

The treatment of cancer draws interest from researchers worldwide. Of the different extracts from traditional Chinese medicines, Tubeimoside 1 (TBMS 1) is regarded as an effective treatment for cancer. To determine the mechanism of TBMS 1, the shape/pattern of HepG2 cells based on the microscopic imaging technology was determined to analyze experimental results; then the fluorescent spectra method was designed to investigate whether TBMS 1 affected HepG2 cells. A three-dimensional (3D) fluorescent spectra sweep was performed to determine the characteristic wave peak of HepG2 cells. A 2D fluorescent spectra method was then used to show the florescence change in HepG2 cells following treatment with TBMS 1. Finally, flow cytometry was employed to analyze the cell cycle of HepG2 cells. It was shown that TBMS 1 accelerated the death of HepG2 cells and had a strong dose- and time-dependent growth inhibitory effect on HepG2 cells, especially at the G2/M phase. These results indicate that the fluorescent spectra method is a promising substitute for flow cytometry as it is rapid and cost-effective in HepG2 cells.

## 1. Introduction

Cancer is one of the most deadly diseases in the world [1]. Effective cancer treatments include surgery [2, 3], radiation therapy [4, 5], chemotherapy [6, 7], gene therapy [8, 9], and biotherapy [10, 11]. However, these methods in addition to killing cancer cells also kill large numbers of normal cells simultaneously. In recent years, the study has drawn significant attention on searching for low toxic antitumor active components from natural plants. Thus, cancer prevention and treatment using Chinese herbal medicines has attracted increasing interest [12, 13]. Chinese herbal medicines have been used in the treatment of different diseases in China and other Asian countries for thousands of years [14]. Biological ingredients of herbal medicines are mainly extracted from natural plants, animal parts, insects, stones, and minerals [15]. In recent decades, numerous basic and clinical studies have been conducted in order to identify effective anticancer agents in Chinese herbal medicines and ascertain their properties in relation to the treatment of cancer. Numerous herbal medicines have been found to have potentially beneficial effects on cancer progression and may ameliorate chemotherapy- or radiotherapy-induced complications and side effects [16, 17].

Primary liver cancer is one of the highest mortality malignant tumors in the world. The incidence of liver cancer is increasing these years. Diagnosis of liver cancer, especially early diagnosis, is essential for improving patient's survival [18]. Thus, it is imminent to seek more effective methods or agents for hepatoma treatment.

Tubeimoside (TBMS), the bulb of Bolbostemma paniculatum (Maxim.) Franquet (Cucurbitaceae), is one of the traditional Chinese medicines often used for the treatment of tumors as well as for detoxication. In traditional Chinese medicine, TBMS has long been widely used for treatment of illness such as inflammation and snake venoms. The potent antitumor activity of TBMS was first reported in 1981. Such antitumor activity in part motivated the successful isolation of Tubeimoside 1 (TBMS 1), a triterpenoid saponin. And subsequent studies confirmed that TBMS 1 can indeed inhibit the growth of several human cancer cell lines including HeLa cells [19, 20] and human promyelocytic leukemia (HL-60) [21]. These studies appear to suggest that TBMS 1 may be a potential candidate as a novel antitumor drug. However, the mechanism of action of TBMS 1 remains unknown [21], even though TBMS 1 is known to preferentially distribute in the liver during* in vivo* metabolism, and thus might better target liver cancer or hepatoma. This study determined whether TBMS 1 affected the growth of cancer cells using the microscopic imaging technology and the fluorescent spectra method. HepG2 cells were selected as the test cells.

Fluorescence spectra of cancer cells can reflect the metabolism status of cancer cells indirectly. To seek the new anticancer medicine and provide useful data for related researches, analyze the result of combination of Tubeimoside and HepG2 cells through fluorescence spectrum technology and the microscopic imaging technology. Substances show a “finger” wave peak due to their fluorescent spectra [22], and this “finger” wave peak is regarded as an identification marker for a particular substance. In this paper, to analyze the consequences of the fluorescent spectra change, the physical specification (shape change) of cells was observed by microscopic imaging technology. The 3D fluorescent sweep was performed to determine the characteristic wave peak of HepG2 cells. Based on the results of the 3D fluorescent sweep, a 2D fluorescent measurement was used to analyze the mechanism of interaction between TBMS 1 and HepG2 cells. To obtain information on the cell cycle, flow cytometry was employed.

## 2. Materials and Methods

### 2.1. Sample Preparation and Cell Culture

As the drug metabolism and pharmacokinetics (DMPK) of the animal models shows that the Tubeimoside 1 is found in highest concentrations in liver, so the Tubeimoside 1 is likely to increase the efficacy of liver cancer. HepG2 cells were obtained from ATCC (American Type Culture Collection) and were cultured at 37°C in 5% CO_2_ in RPMI-1640 (Thermo Fisher Scientific Inc., USA) plus 10% fetal bovine serum (Zhejiang Tianhang Biological Technology Co., Ltd., China) under standard culture conditions. HepG2 cells should be gotten through the primary culture and subculture. Then we took out three bottles of cells from the cell culture incubator, observed, recorded the growth condition of cultured cells, replaced the RPMI-1640 culture timely, repeated the subculture when the density of the cells in flasks was larger, and subpacked each bottle of cells in 2 bottles; the six bottles of cells were labeled (a)–(f), and then we put them back to the cell culture incubator. When the cells reached 60%–70% confluence, they were subcultured or treated with TBMS 1.

TBMS 1 (National Institute for Food and Drug Control, China) was dissolved in PBS and stored at 4°C and then diluted to 15 *μ*mol/L and 30 *μ*mol/L, respectively. HepG2 cells were treated with TBMS 1 at these two concentrations for 24 hours and 48 hours. Untreated cells were considered as the control group, and 6 groups of samples were analyzed in total. These groups were labeled (a)–(f) and are given in Table 1. To eliminate interference caused by different concentrations of HepG2 cells and avoid saturation of the fluorescent intensity, 4 mL of the cell suspension was withdrawn and then diluted to approximately 10000 counts/mL for the fluorescent measurement, while the remainder was used for flow cytometry.

### 2.2. Microscopic Imaging of HepG2

To analyze the interaction between TBMS 1 and HepG2 cells according to the fluorescent spectra, microscopic images of these cells after treatment were gotten using a microscope (Leica DMI6000B, Germany) with a 40x objective lens and a 10x eyepiece; then the pattern changes were observed by the comparative analysis of the microscopic images.

### 2.3. Fluorescent Measurement

This experimental section firstly studied the general characteristics of each HepG2 cells group before and after the interaction with Tubeimoside 1 by using three-dimensional fluorescence spectrum technique, then studied the detail characters by using the two dimensional fluorescence spectrum. By comparing the fluorescence spectrum of each HepG2 cells group, we analysed the difference between each other and got the experimental conclusion.

To acquire the fluorescent characteristics of the HepG2 cells, a 3D fluorescent spectra sweep of group (a)–(c) was performed using a fluorospectrophotometer (F97, Shanghai Lengguang Technology Co., Ltd., China). The excitation light was set at 200 nm to 700 nm with a resolution of 20 nm. The emission light was also set at 200 nm to 700 nm with a resolution of 1 nm. The sweep speed was placed at 6000 nm/min. Before testing, and to ensure the samples were stable, a xenon lamp was shone on the samples for 3 minutes and the temperature controller on the fluorospectrophotometer was turned on for half an hour. Three tests were performed for each sample and the average value was calculated for analysis.

Based on the 3D fluorescent spectra, the wavelength of the characteristic emission peak was determined. The wavelength of the excitation light was then set. The 2D fluorescent spectra were then acquired. Three tests were carried out for each sample and the average value was calculated for analysis.

### 2.4. Flow Cytometry for Determination of Cell Cycle Distribution

This experiment analysed the characteristics of HepG2 cells changes before and after the interaction with Tubeimoside 1. After the fluorescent measurements, the remaining cells in each group were harvested and analyzed using flow cytometer (BD FACSCalibur, BD Biosciences, USA). The main purpose was to determine the cell cycle distribution of the HepG2 cells before and after the interaction with Tubeimoside to analyze the results of the fluorescence spectrum experiment.

## 3. Results and Discussion

### 3.1. Analysis of HepG2 Cells Images

**Figure 1:**
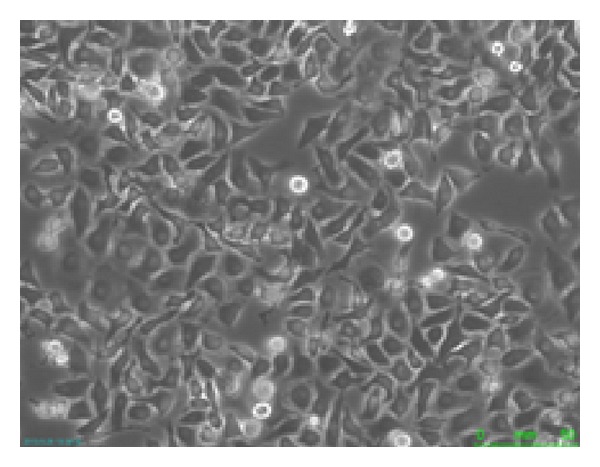
Cell morphology of HepG2 cells before adding the Tubeimoside 1.

Before adding the Tubeimoside 1 to the cell culture bottles of HepG2 cells, we got the image of the HepG2 cells shown in Figure 1, the magnification of the microscope was ×400.

**Figure 2:**
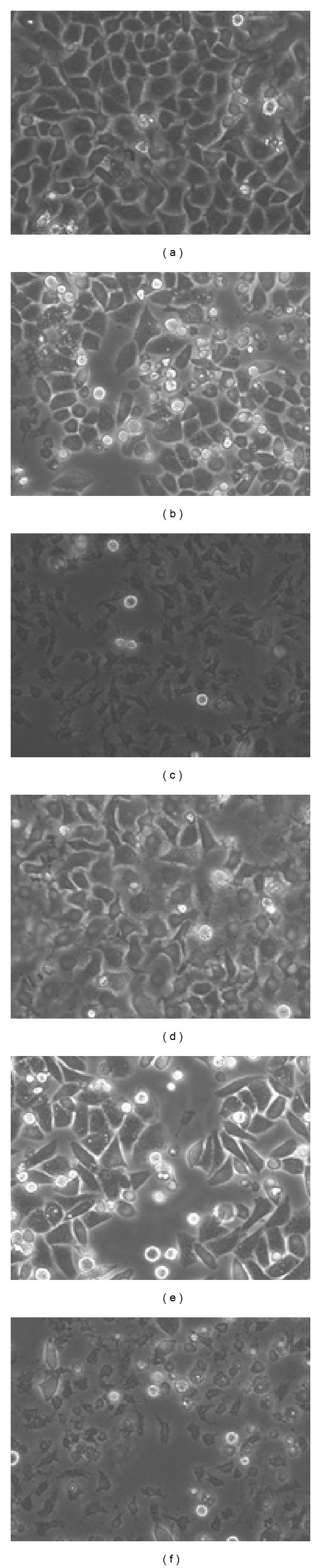
Photographs of the 6 groups of HepG2 cells.

When the Tubeimoside 1 had been in the cell cultures for 24 hours, the cell morphology images of groups (a)–(c) were shown in Figures 2(a)–2(c). When the Tubeimoside 1 had been in the cell cultures for 48 hours, the cell morphology images of groups (d)–(f) were shown in Figures 2(d)–2(f).

From images of recorded cell morphology at various points, we can see that Tubeimoside 1 had significant effects on HepG2 cells. From Figures 2(b) and 2(c), the cells were more dispersed and were round in shape. Figures 2(c) and 2(d) showed numerous dead cells. In Figures 1, 2(a), and 2(c), the membrane of HepG2 cell had the vesicles bulging outward gradually; the phenomenon of apoptosis was obvious; and a lot of cells died. So it could be got that the effects that Tubeimoside 1 had on HepG2 cell were more obvious along with the increasing of concentration of Tubeimoside 1. And the longer the time of the interaction between Tubeimoside 1 and HepG2 cells was, the better the effects were.

In a word, TBMS 1 accelerated the death of HepG2 cells and this acceleration was proportional to the concentration of tubeimoside1 and duration of treatment.

### 3.2. The Survival Rate of HepG2 Cell

**Figure 3:**
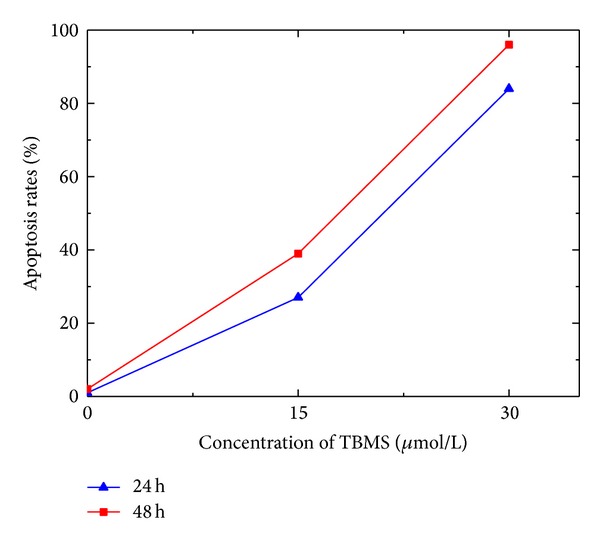
The apoptosis rate of HepG2 cells.

After adding the Tubeimoside 1, HepG2 cells began to die in different degrees. Due to limited experimental conditions. We counted the apoptosis rate of each group of cells based on the images taken by using the electron microscope. Ten visual field images of each group of cells were selected from the microscopic images of 400, then calculated the average of the cell apoptosis rates, respectively. The result was shown in Figure 3.

By means of statistics analysis, the apoptosis rates of groups (a)–(c) were 1%, 27%, and 84%, respectively, and the apoptosis rates of groups (d)–(f) were 2%, 39%, and 96%, respectively.

### 3.3. Fluorescent Change

This experiment firstly measured the three-dimensional fluorescence spectrum of each group of HepG2 cell suspension, then determined the general position of the fluorescence peak and exported the data and recorded the corresponding emission wavelength of fluorescence peak. Depending on the recorded emission wavelength, we measured the excitation spectrum of each group, then determined the fluorescence peak position from the excitation spectrum and recorded the corresponding excitation wavelength. Finally, we measured the corresponding emission spectrum according to the records of the excitation wavelength, and the emission spectrum was the detailed information of the three-dimensional fluorescence spectrum peak.

**Figure 4:**
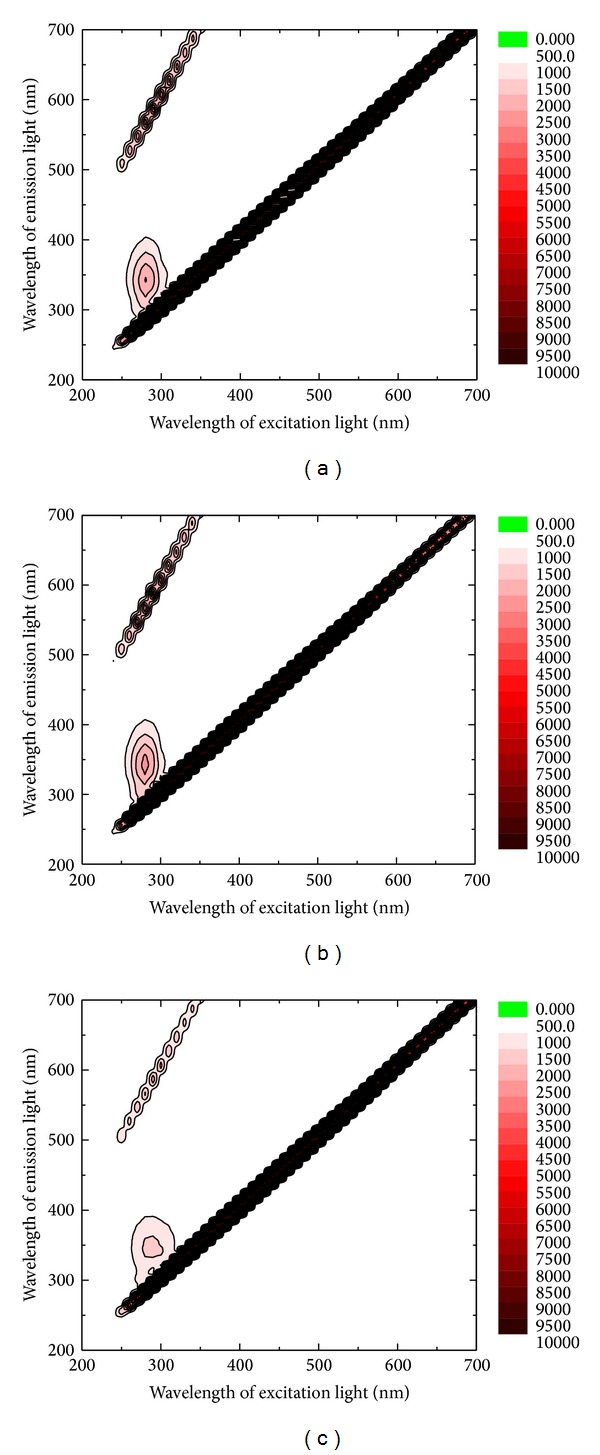
3D fluorescent spectra of groups (a)–(c).

Figure 4 shows the 3D fluorescent spectra of the sample groups (a)–(c). Except for Raman and Rayleigh scattering, the strongest intensity of emitted light was seen when the excitation light was approximately 282 nm. It was concluded from Figure 4 that the contour plot of three-dimensional fluorescence peaks was more and more sparse and the relative fluorescence intensity was decreasing to various degrees with the increase in the concentration of the Tubeimoside 1.

**Figure 5:**
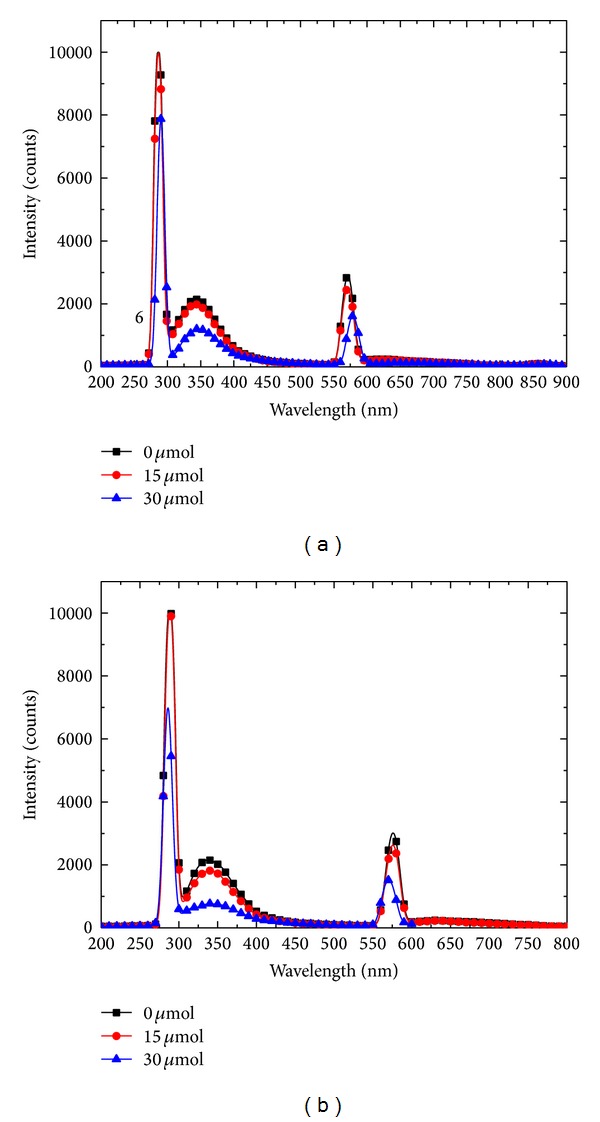
Emission spectra of the 6 groups of HepG2 cells with excitation light at 282 nm.

According to the 3D fluorescent spectra, the wavelength of excitation light was set at 282 nm.  Figure 5 shows the 2D fluorescent spectra of emitted light at wavelengths ranging from 200 nm to 800 nm.

The intensity of the emitted light near 342 nm in groups (a)–(f) was 2154, 1997, 1196, 2162, 1819, and 765, respectively. It was concluded that the intensity of the characteristic peak of HepG2 cells was weak when the concentration of TBMS 1 was high. The longer the treatment period is, the weaker the intensity of the characteristic peak of HepG2 appeared. 340 nm was the characteristic peak position and may have been caused by tryptophan [23].

The intensity of the Rayleigh scattering near 282 nm in groups (a)–(f) was 10000, 9976, 7871, 10000, 9959, and 6994, respectively. Similarly, with increasing concentration of TBMS 1 the intensity of the characteristic peak decreased.

It appears that Tubeimoside 1 could not only influence the position of HepG2 cells fluorescence peak, but also affect the relative light intensity. The intensity of the characteristic peak at 342 nm changed with the concentration of TBMS 1. The relative light intensity at fluorescence spectrum peak was inversely related to concentration of Tubeimoside 1 and duration of treatment. The possible reason was that Tubeimoside 1 influenced the distribution of HepG2 cells cycle phase and caused the apoptosis of HepG2 cells.

### 3.4. Results of Flow Cytometry

The influence of Tubeimoside 1 on the cell cycle distribution was measured by detecting the cellular DNA content using the flow cytometry.  Table 2 shows the results of flow cytometry in the samples.

By comparing the experimental results of groups (a)–(c), it was not difficult to see that after 24 hours interaction between Tubeimoside 1 and HepG2 cells, the ratio of HepG2 cells in G1 phase decreased gradually while the HepG2 cells in G2-M phase and S phase increased gradually with the increase of the concentration of Tubeimoside 1. Similarly, we also compared the flow cytometry experiment results of groups (d)–(f). After 48 hours interaction between Tubeimoside 1 and HepG2 cells, the ratio of HepG2 cells in G1 phase decreased gradually while the HepG2 cells in G2-M phase increased gradually with the increase of the concentration of Tubeimoside 1, but the HepG2 cells in S phase had not the similar variation. For the HepG2 cells in G2-M phase, by comparing group A with group D, group B with group E, and group C with group F; respectively, it was concluded that the number of cells was directly proportional to the time of interaction between Tubeimoside 1 and HepG2 cells when the concentration of Tubeimoside 1 was the same. It could also be concluded that the difference values between group A and group D, group B and group E, and group C and group F were increasing with the increase of the concentration of Tubeimoside 1. The above results showed that TBMS 1 had a growth inhibitory effect on HepG2 cells, particularly in the G2/M phase.

## 4. Conclusion

This paper analyzed the cell morphological microscopic image changes, the three-dimensional fluorescence spectrum and the emission spectrum changes, and flow cytometry results of the HepG2 cells before and after the interaction with the Tubeimoside 1, respectively.

The apoptosis of HepG2 cells can be measured by microscopic image, and the information also can be reflected by the fluorescence spectrum. In other words, fluorescence spectrometry probably can be used as a detection method of HepG2 cell apoptosis. Conventional method of cell apoptosis is using flow cytometry, but the operation process is complex and the experiment cost is high compared with the fluorescence spectrum analysis method. So these results also indicate that the fluorescent spectra method is a promising substitute for flow cytometry as it is rapid and cost-effective in HepG2 cells.

## Figures and Tables

**Table 1:** Preparation of samples for assay.

Group ID	A	B	C	D	E	F
Concentration of TBMS 1 (*μ*mol/L)	0	15	30	0	15	30
Duration of Treatment (hours)	24	24	24	48	48	48

**Table 2:** The results of flow cytometry.

Sample ID	A	B	C	D	E	F
Proportions of cells in G1 phase (%)	67.56	63.32	55.88	45.68	51.72	41.15
Proportions of cells in S phase (%)	24.99	27.53	33.77	39.59	27.38	35.19
Proportions of cells in G2/M phase (%)	7.45	9.15	10.34	14.73	20.90	23.66
